# Caregiver satisfaction with paediatric HIV treatment and care in Nigeria and equity implications for children living with HIV

**DOI:** 10.1080/09540121.2016.1176682

**Published:** 2016-07-08

**Authors:** Dick Chamla, Chukwuemeka Asadu, Ebun Adejuyigbe, Abiola Davies, Ebele Ugochukwu, Lawal Umar, Ilesanmi Oluwafunke, Fatimah Hassan-Hanga, Chinyere Onubogu, Immaculata Tunde-Oremodu, Chinelo Madubuike, Esther Umeadi, Obed Epundu, Adenike Omosun, Emmanuel Anigilaje, Daniel Adeyinka

**Affiliations:** ^a^ Health Section, UNICEF, New York, NY, USA; ^b^ NASCP, Federal Ministry of Health, Abuja, Nigeria; ^c^ Faculty of Clinical Sciences, Obafemi Awolowo University, Ile-Ife, Nigeria; ^d^ Country Office, UNICEF, Abuja, Nigeria; ^e^ Department of Paediatrics, Nnamdi Azikiwe University Teaching Hospital, Nnewi Anambra State, Nigeria; ^f^ Department of Paediatrics, ABU Teaching Hospital Zaria, Kaduna State, Nigeria; ^g^ World Health Organization, Abuja, Nigeria; ^h^ Paediatrics Department, Aminu Kano Teaching Hospital/Bayero University, Kano State, Nigeria; ^i^ Paediatric HIV and Infectious Disease Unit, Federal Medical Centre, Yenagoa Bayelsa State, Nigeria; ^j^ Health Service Commission, Lagos state, Nigeria

**Keywords:** Caregiver, satisfaction, paediatric HIV, equity

## Abstract

Caregiver satisfaction has the potential to promote equity for children living with HIV, by influencing health-seeking behaviour. We measured dimensions of caregiver satisfaction with paediatric HIV treatment in Nigeria, and discuss its implications for equity by conducting facility-based exit interviews for caregivers of children receiving antiretroviral therapy in 20 purposively selected facilities within 5 geopolitical zones. Descriptive analysis and factor analysis were performed. Due to the hierarchical nature of the data, multilevel regression modelling was performed to investigate relationships between satisfaction factors and socio-demographic variables. Of 1550 caregivers interviewed, 63% (95% CI: 60.6–65.4) reported being very satisfied overall; however, satisfaction varied in some dimensions: only 55.6% (53.1–58.1) of caregivers could talk privately with health workers, 56.9% (54.4–59.3) reported that queues to see health workers were too long, and 89.9% (88.4–91.4) said that some health workers did not treat patients living with HIV with sufficient respect. Based on factor analysis, two underlying factors, labelled *Availability* and *Attitude*, were identified. In multilevel regression, the satisfaction with availability of services correlated with formal employment status (*p* < .01), whereas caregivers receiving care in private facilities were less likely satisfied with both availability (*p* < .01) and attitude of health workers (*p* < .05). State and facility levels influenced attitudes of the health workers (*p* < .01), but not availability of services. We conclude that high levels of overall satisfaction among caregivers masked dissatisfaction with some aspects of services. The two underlying satisfaction factors are part of access typology critical for closing equity gaps in access to HIV treatment between adults and children, and across socio-economic groups.

## Introduction

Globally, there are over 2.4 million children living with HIV who do not receive HIV treatment and care (UNAIDS, 2014b). Most of these children reside in sub-Saharan Africa where a persistent gap in access to antiretroviral therapy (ART) between adults and children constitutes a major inequity in the global HIV response (UNAIDS, 2014b). In 2014, less than one-third of children living with HIV received life-saving ART compared to 40% for adults and 66% for pregnant women among the 21 Global Plan priority countries (UNAIDS, 2014a). In Nigeria, only 12% of children living with HIV receive treatment and care which is among the lowest rates of ART coverage in the world (UNAIDS, 2014a, 2014b). Prominent among the factors related to this low coverage are limitations in policy and health systems infrastructure, such as limited decentralization of services, inadequate child-friendly Antiretroviral (ARV) formulations and ineffective case-finding strategies (McNairy et al., 2013). Most of these factors to date have focused primarily on the supply side (care provision) with little consideration for the demand side, for instance, caregivers’ perspectives on the quality of paediatric HIV services.

Prior studies indicate that primary caregivers play a major role in recognizing children’s needs and ensuring that their children enter and remain in health care (Cook et al., 2004; Logan & King, 2002). Parents’ satisfaction with the health services their children receive has been shown to influence utilization, length of service usage and to enhance relationships with healthcare providers (Cook et al., 2004). In the health sector, the definition of client satisfaction is anchored to the fulfilment of the individual’s desire for and expectations of health care (Kravitz, 1998). Zemencuk, Feightner, Hayward, Skarupski, and Katz (1998) defined the patients’ desires as their perception of needing a given element of care. It is increasingly recognized that viewing health care “through patients” eyes’ is ethically and professionally imperative as the core purpose of health systems is to also help patients achieve their desires and expectations (Kravitz, 1998). In Nigeria, caregiver satisfaction with non-ART-related child health services was previously reported to vary among public health facilities (Iloh et al., 2013). However, in the provision of paediatric HIV services, there is a paucity of literature on caregiver satisfaction with HIV services for their children. This is one of the missing links for fully understanding the drivers of low coverage of paediatric ART and poor retention.

Few studies in health care have examined the relationship between caregiver satisfaction and equity in HIV care. In the business sector, however, many such studies abound with diverse findings on the relationship between customer satisfaction and equity (Fisk & Young, 1985). In developed countries, several studies have reported that utilization of health services in general hospital settings, after controlling for healthcare needs, promoted horizontal equity – meaning equal access for equal needs regardless of socio-economic differences (Lostao, Blane, Gimeno, Netuveli, & Regidor, 2014). Building on these studies, it can be postulated that satisfied caregivers are likely to improve utilization of services for their children living with HIV irrespective of their socio-economic differences resulting in equitable treatment access and outcomes.

In this study, we measured dimensions of caregiver satisfaction with HIV care services provided to their HIV-positive children in Nigeria, and assessed the relationship of underlying satisfaction factors with caregiver characteristics. To the best of our knowledge, this is the first study that assesses caregiver satisfaction with paediatric HIV and care. We anticipate that the findings may contribute significantly to the existing literature. The aim was to understand the implications of caregiver satisfaction on equity for their children and suggest how their perspectives on paediatric HIV treatment could be addressed within national HIV strategic planning and policy dialogues.

## Methods

The study was multi-centre and facility-based that included exit interviews for caregivers of children living with HIV and receiving ART in selected facilities in Nigeria. This was conducted as part of larger paediatric HIV assessment supported by United Nations Children Fund and the World Health Organization. It was carried out in collaboration with the HIV/AIDS Division of the Federal Ministry of Health and members of the Paediatric sub-Committee of the National ART Task Team drawn from the academia and partner organizations. The eligibility criteria included a primary caregiver (a parent or member of the family) residing with and caring for the child aged less than 15 years living with HIV who visited the ART facility for a scheduled clinical appointment. ART facilities routinely kept updated appointment registers and folders containing medical records of children receiving HIV treatment and care. Based on Nigeria’s ART guidelines, the clinical appointments are scheduled to monitor clinical and immunological response to ART, screen for tuberculosis, track adherence, monitor adverse drug reactions and relevant laboratory parameters depending on each client’s condition.

### Sampling and data sources

Purposive sampling was used to select a total of 20 public and private facilities offering paediatric ART services from 5 states (Anambra, Bayelsa, Benue, Kano and Lagos). These represented a mixture of high and low HIV burden in five geopolitical zones. The list of facilities was drawn from the Nigerian Health Facility Directory, and sorted by key domains for wide representation. These included urban/rural location, and facility level and ownership (public/private [for-profit and not-for-profit such as faith-based]).

We used records of clinic appointments from the selected facilities to estimate the number of caregivers to be enrolled for exit interviews. Using PASS 12 software^®^, (NCSS, Kaysville, Utah, USA, 2012) and adjusting for 20% non-response rate, a sample size of 1500 caregivers was required. This was then distributed proportionally to the population size of the facilities based on the number of patients ever enrolled in paediatric HIV treatment and care. A systematic selection of caregivers was done in each facility by randomly selecting the first caregiver using Excel “Rand” function followed by every third individual in the appointment register.

A structured questionnaire adapted from the 13-item patient satisfaction tool (Chimbindi, Barnighausen, & Newell, 2014) was used to collect information for assessing caregiver satisfaction with paediatric HIV treatment focusing on perceptions and evaluation of treatment received. This patient questionnaire has been validated in sub-Saharan African settings and has been used previously for patients receiving HIV treatment (Lyatuu, Msamanga, & Kalinga, 2008; Paddock, Veloski, Chatterton, Gevirtz, & Nash, 2000). The adaptation involved the caregivers’ responses regarding HIV treatment on behalf of their children. The questions were adapted to elicit caregiver perceptions on overall satisfaction, staff–caregiver communications, staff attitudes, privacy, confidentiality, staffing and amenities. The adaptation of the patient questionnaire used for caregivers was based on the premise that primary caregivers, particularly parents, have major role in recognizing their children’s needs and have strong impact on children’s use of ART medication. It was also presumed that caregiver perceptions and expectations of services provided to their children do have major influence on access and adherence to paediatric HIV treatment.

### Outcomes and data analysis

The main outcome of the study was caregiver satisfaction and its underlying factors. Socio-demographic data associated with satisfaction factors included age, sex, marital status, level of education, employment, income status and facility ownership. Most of these are individual patient-level factors that have been found to influence patient satisfaction in previous studies (Quintana et al., 2006).

Descriptive analysis of the dimensions of caregiver satisfaction was performed, followed by a factor analysis with oblique rotation using the Promax method to identify underlying factors that explain satisfaction data. The oblique rotation was used based on the theoretical assumptions that the factors for patient satisfactions are correlated. In line with Kaiser criteria, those factors with eigenvalue >1.00 were retained after factor analysis. The rotated factor loadings were tabulated and a Scree plot was generated to visualize those factors with eigenvalue greater than 1.00. Retained factors were labelled based on the weights and number of loadings. Additionally, due to the hierarchical nature of data collected, of individuals nested within facilities and states, we performed a multilevel regression modelling to establish the relationship between the satisfaction factors identified and socio-demographic variables at statistical significance of *α* < 0.05. Random-effect equations were used to measure the variations at facility and state levels. All statistical analyses were completed using STATA version IC 11 (StataCorp LP, College Station, TX, USA, 2012).

### Ethical considerations

Ethical clearance for the study was granted by National Health Research Ethics Committee of Nigeria (NHREC). Formal requests and approvals to visit facilities and perform exit interviews were also granted by the Federal and State Ministries of Health. Informed consents signed by the study participants were obtained before the exit interviews. Confidentiality of information from the caregivers was assured by removal of personal identifiers.

## Results

A total of 1550 caregivers were interviewed, of which 1386 (89.4%) attended public facilities and 973 (62.8%) attended tertiary-level facilities providing paediatric HIV treatment and care. The socio-demographic characteristics of the caregivers are presented in Table 1. Most caregivers were females (1333 [86.1%]) with the median age of 33 years [interquartile range (IQR): 28–39], married, and over 62% earned less than NGN 18,000 per month.

**Table 1.** Socio-demographic characteristics of caregivers.

Variable	*N*	%
Age in years (*n* = 1533)		
15–19	15	1
20–29	455	29.7
30–39	710	46.3
40–49	242	15.8
50 and above	111	7.2
Sex (*n* = 1549)		
Female	1333	86.1
Caregiver relationship to a child (*n* = 1546)		
Mother	1257	81.3
Father	130	8.4
Relative	159	10.3
Marital status (*n* = 1546)		
Married	1210	78.3
Single, separated or divorced	179	11.5
Widow or widower	157	10.2
Education level (*n* = 1546)		
None or informal	170	11.0
Primary	285	18.4
Secondary	703	45.5
Postsecondary Diploma and above	388	25.1
Employment status (*n* = 1532)		
None	326	21.3
Employed in informal sector	988	64.5
Employed in formal sector	218	14.2
Income status (*n* = 1474)		
<N18,000	914	62.0
N18,000–50,000	427	29.0
N51,000–100,000	105	7.1
>N100,000	28	1.9

In total, 63.0% (95% CI: 60.6–65.4%) of caregivers were overall very satisfied, but the level of satisfaction among particular dimensions varied widely as depicted in Table 2.

**Table 2.** Dimensions of caregiver satisfaction.

Domains of caretaker satisfaction	Question	Response	*N*	% (95% CI)
1. Health worker–caregiver communication	(a) Health workers discussed the treatment of my child fully with me	Agree	1537	93.8 (92.6–95.0)
	(b) I find it easy to tell the health worker when I have missed giving medication to my child	Agree	1538	67.8 (65.4–70.1)
	(c) It is a problem that health workers do not speak my language	Agree	1542	7.7 (6.3–9.0)
	(d) The health workers are too busy to listen to the problems of my child	Agree	1547	19.4 (17.4–21.4)
2. Health worker attitude	(a) Some health workers do not treat patients who are living with HIV with sufficient respect	Agree	1543	89.9 (88.4–91.4)
	(b) The health workers I have seen respect me	Agree	1543	93.8 (92.6–95.0)
3. Privacy and Confidentiality	(a) Patient information is kept confidential in this facility	Agree	1540	90.0 (88.5–91.5)
	(b) In this facility you are able to talk to the doctors or nurses in private	Always	1532	55.6 (53.1–58.1)
4. Staffing and services	(a) The queues to see the health worker for paediatric HIV services are too long at this facility	Agree	1544	56.9 (54.4–59.3)
	(b) For your child ARV treatment, what would you prefer?	See a nurse in nearby facility	1471	57.3 (54.8–59.8)
		Travel further to see doctor	1471	32.2 (29.8–34.6)
	(c) How do you think the service in this facility could be improved?	(a) Shorter queues	1462	81.7 (79.7–83.7)
		(b) More health workers	1460	80.2 (78.2–82.3)
		(c) Cleaner facilities	1416	60.7 (58.1–63.2)
		(d) Better facilities	1354	69.8 (67.3–72.2)
Overall satisfaction	How satisfied were you with services for your child today?	Very satisfied/satisfied	1547	63.0 (60.6–65.4)

### Communication and health worker attitude

Most respondents (93.8%) reported that health workers fully discussed HIV treatment of their children with them; yet, only two-thirds found it easy to tell the health workers if they missed giving medication to their children. Despite the majority disagreeing that it was a problem that health workers did not speak caregivers’ language, approximately one-fifth thought that health workers were too busy to listen to their children’s problems. Similarly, almost 90% of caregivers agreed that some health workers did not treat patients living with HIV with sufficient respect. In contrast, almost 94% of respondents agreed that they, as caregivers, were treated with respect.

### Privacy, confidentiality, staffing and services

The majority of respondents (90%) agreed that patient information was kept confidential in the health facilities they visited; however, only 55.6% reported that they were able to talk with the doctors or nurses in private. Additionally, approximately 57% of respondents admitted that the queues to see a health worker for services were too long. A similar proportion (57.3%) would have preferred to see a nurse in a nearby clinic than to travel further to see a doctor (32.2%) to access HIV services for their children. In order to improve services in the health facilities, most respondents suggested shorter queues (81.7%), making more health workers available to clients (80.2%), making health facilities cleaner (60.7%) and provision of better patient amenities such as waiting rooms or toilets (69.8%).

**Figure 1.**
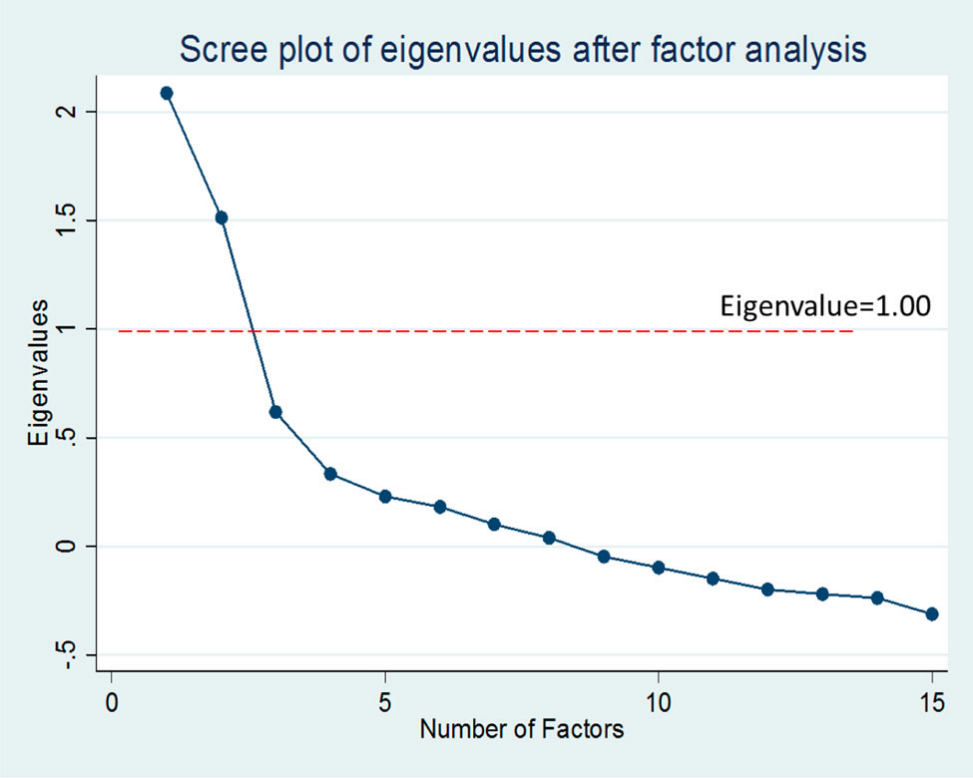
Scree plot of eigenvalues.

In the factor analysis of caregiver satisfaction, two underlying factors with eigenvalue above 1.00 were retained (Figure 1). These two factors accounted for 93.8% of total variance in caregiver satisfaction. Table 3 shows the factor loadings for each satisfaction variable. Based on factor loadings, we labelled Factor 1 as *availability* of services and Factor 2 as *attitude* of health workers. The labels capture the contents of different satisfaction variables that load heavily on the two retained factors.

**Table 3.** Factor loadings after oblique rotation.

Variables	Availability	Attitude	Uniqueness
How satisfied were you with services for your child today?	−0.0259	0.5446	0.6992
Health workers discussed the treatment of my child fully with me	−0.0021	0.3366	0.8865
I find it easy to tell the health worker when I have missed giving medication to my child	0.3166	0.1003	0.8977
It is a problem that health workers do not speak my language	0.1075	−0.1795	0.9514
The health workers are too busy to listen to the problems of my child	0.0175	−0.2474	0.9374
Some health workers do not treat patients who are living with HIV with sufficient respect	0.0422	0.6806	0.5423
The health workers I have seen respect me	−0.0107	0.6047	0.6326
Patient information is kept confidential in this facility	0.0024	0.2892	0.9165
The queues to see the health worker for paediatric HIV services are too long at this facility	0.1012	−0.2049	0.9425
In this facility, you are able to talk to the doctors or nurses in private	0.1783	0.3163	0.8824
For your child’s ARV treatment, what would you prefer: To travel further to see doctor = Yes	−0.0663	−0.0716	0.9917
How do you think the service in this facility could be improved?			
(a) Shorter queues	0.5482	−0.1241	0.6668
(b) More health workers	0.5872	−0.0418	0.6472
(c) Cleaner facilities	0.8164	0.0627	0.3426
(d) Better patient facilities (toilets, waiting room area)	0.7317	0.0160	0.4673

In multilevel regression modelling (Table 4), the level of satisfaction with availability of services had a positive association with formal employment status (*p* < .05), whereas those caregivers who received care in private facilities were less likely to be satisfied with both availability of services (*p* < .01) and attitude of health workers (*p* < .05). Analysis of random-effect parameters was used to measure the variations at facility and state levels and showed that states and facility levels did not influence the association between the levels of satisfaction with availability of services (*p* > .05), but had significant association with the attitude of the health workers (*p* < .01). The level of satisfaction was not associated with age, sex, marital status or income.

**Table 4.** Socio-demographic factor associated with caregiver satisfaction.

	Availability			Attitude		
	Coef.	95% CI	*p* value	Coef.	95% CI	*p* value
Sex: female	0.10	−0.15–0.34	.45	0.01	−0.19–0.22	.89
Age	−0.005	−0.01–0.00	.09	−0.003	−0.01–0.002	.21
Marital status: *married*	0.08	−0.05–0.21	.23	−0.04	−0.15–0.06	.43
Relationship with a child:						
Mother	−0.02	−0.21–0.18	.86	−0.12	−0.28–0.04	.14
Father	0.11	−0.19–0.41	.49	−0.05	−0.29–0.20	.70
Education: *secondary and above*	−0.09	−0.22–0.03	.12	−0.02	−0.12–0.08	.70
Employment: *formal employee*	0.25	0.10–0.41	.002	0.11	−0.02–0.24	.11
Annual income status:						
<N18,000	0.05	−0.36–0.46	.81	−0.05	−0.38–0.28	.76
N18,000–50,000	−0.22	−0.62–0.19	.30	−0.03	−0.37–0.30	.84
N51,000–100,000	−0.09	−0.53–0.35	.70	−0.07	−0.43–030	.72
>N100,000 (ref)						
Facility ownership: *private*	−0.32	−0.51–(−0.13)	.001	−0.18	−0.34–(−0.30)	.03
Random-effects parameters						
State: sd(_cons)	0.021	1.1e^-12^–3.9e^+8^		0.28	0.05–1.43	
Facility level: sd(_cons)	0.093	0.02–0.37		0.16	0.05–0.47	
sd (residual)	0.876	0.84–0.91		0.72	0.69–0.75	
LR test vs. linear regression	*p* = .1569			*p* = .0000		

## Discussion

Caregiver satisfaction with paediatric HIV services has the potential to influence access and outcomes of HIV, hence the importance of this study. In Nigeria, provision of high-quality care for children living with HIV was shown to improve outcomes by lowering loss to follow-up and decreasing mortality (Ojikutu et al., 2014). Despite this, there is limited evidence on the utility of caregiver satisfaction data in policy formulations in sub-Saharan Africa.

Our study found that approximately two-thirds of caregivers, while very satisfied overall with treatment offered to their children living with HIV, showed substantial variations in the levels of satisfaction regarding aspects of staff attitude, privacy, availability of services and waiting time to see health workers due to the long queues. This is consistent with previous studies that indicated that a high level of overall satisfaction masked substantial dissatisfaction with particular aspects of services (Chimbindi et al., 2014). It also confirmed findings of other studies that found waiting times due to long queues to be a main determinant of patient satisfaction (Dansky & Miles, 1997).

The finding that most caregivers preferred a nurse in a nearby facility rather than travelling further to see a doctor supports the need for a national policy on decentralization of paediatric HIV treatment and care in Nigeria. At the time of this study, paediatric ART was initiated only at secondary or tertiary levels of care. The potential effect of decentralization of services on increasing access to paediatric HIV services has been well documented (Fayorsey et al., 2013). Similarly, caregivers overwhelmingly preferred shorter queues and providing additional health workers, highlighting important strategies for improving paediatric HIV services. This is consistent with a study in Bangladesh that found a reduction in waiting time as more important to clients seeking for healthcare services (Mendoza Aldana, Piechulek, & al-Sabir, 2001). A preference for more health workers also reflects the limitation of current policy in Nigeria that only allows initiation of paediatric HIV treatment by medical doctors or specialist paediatricians. This underscores the need for task shifting in paediatric HIV services that could improve HIV services as observed in other countries (Kredo, Adeniyi, Bateganya, & Pienaar, 2014).

This study identified *availability* and *attitude* as the main factors underlying caregiver satisfaction. These factors are in agreement with two of the five dimensions of “access” taxonomy, proposed by Penchansky and Thomas (1981). Later in 1984, Thomas and Penchansky described the relationship of satisfaction with access to services, further supporting the hypothesis that patients’ beliefs and perception are important determinants of health behaviour. Thus, the low levels of caregiver satisfaction with availability and attitude observed in this study might partly explain the reported low coverage of paediatric HIV treatment in Nigeria. The findings in this study are also consistent with other studies that identified provider behaviour, particularly respect and politeness, as the most powerful predictors of client satisfaction (Mendoza Aldana et al., 2001).

This study also demonstrated that only formal employment and receiving services in private health facility influenced the two underlying factors. A meta-analysis reported by Hall and Dornan (1990) concluded that socio-demographic characteristics are at best a minor predictor of satisfaction. Other studies have also shown inconsistent relationships between patient socio-demographic characteristics and satisfaction (Jackson, Chamberlin, & Kroenke, 2001). The finding of a positive relationship between formal employment and the level of satisfaction on availability of services may be explained by the fact that formal employees are participants of the National Health Insurance Scheme (NHIS) that covers parents and up to 4 children under the age of 18 years in Nigeria (Mohammed, Sambo, & Dong, 2011). Similarly, other studies in Nigeria have indicated that formal employment was a strong predictor of willingness to pay for HIV care, as out-of-pocket payments in health is over 90% (Mbachu, Enabulele, Nwudele, Alegu, & Anwara, 2015; Odeyemi & Nixon, 2013). The inverse relationship between satisfaction and private facilities is likely due to unmet expectation of caregivers for the services in private facilities. This finding is in line with the “discrepancy model” of patient satisfaction by Fox and Storms (1981) arguing that satisfaction is entirely relative, and defined in large part by the perceived discrepancy between a patient’s expectation and actual experience.

The findings on caregiver satisfaction have implications for the equity for children living with HIV receiving life-saving treatment. We have demonstrated that the underlying satisfaction factors, *availability* and health workers *attitude*, are critical dimensions of access to services. Improving access to paediatric HIV services is essential for closing the equity gap in access to ART between adults and children. The improved access and retention will likely improve equitable treatment outcomes between rich and poor, as income status did not influence our satisfaction data. Improved access across population groups will also reduce the possibility of “inverse inequity”, as suggested by Cesar Victora, where wealthier individuals benefit first from new and better interventions (Hargreaves, Davey, & White, 2013). In this study, we did not investigate the relationship between satisfaction and treatment outcomes – a future research question requiring further elucidation.

We also hypothesized that the influence of formal employment on satisfaction is most likely due to the access to Health Insurance Scheme which extends the coverage of care to children. This re-emphasizes the importance and need for universal health coverage, which has been shown to promote equity (Atun et al., 2013). Conversely, studies in the business sector have also shown that the relationship between satisfaction and equity is bi-directional with evidence indicating that perceived unfairness in service provision has led to customer dissatisfaction (Fisk & Young, 1985). A limitation of this study, however, is that we did not determine whether there were differences in the quality of services provided by selected facilities or states even though the levels of facility and state had significant influence on the attitude of health workers.
